# Continuous versus Cyclic Progesterone Exposure Differentially Regulates Hippocampal Gene Expression and Functional Profiles

**DOI:** 10.1371/journal.pone.0031267

**Published:** 2012-02-29

**Authors:** Liqin Zhao, Todd E. Morgan, Zisu Mao, Sharon Lin, Enrique Cadenas, Caleb E. Finch, Christian J. Pike, Wendy J. Mack, Roberta D. Brinton

**Affiliations:** 1 Department of Pharmacology and Pharmaceutical Sciences, School of Pharmacy, University of Southern California, Los Angeles, California, United States of America; 2 Davis School of Gerontology, University of Southern California, Los Angeles, California, United States of America; 3 Department of Preventive Medicine, Keck School of Medicine, University of Southern California, Los Angeles, California, United States of America; 4 Department of Neurology, Keck School of Medicine, University of Southern California, Los Angeles, California, United States of America; Catholic University Medical School, Italy

## Abstract

This study investigated the impact of chronic exposure to continuous (CoP4) versus cyclic progesterone (CyP4) alone or in combination with 17β-estradiol (E2) on gene expression profiles targeting bioenergetics, metabolism and inflammation in the adult female rat hippocampus. High-throughput qRT-PCR analyses revealed that ovarian hormonal depletion induced by ovariectomy (OVX) led to multiple significant gene expression alterations, which were to a great extent reversed by co-administration of E2 and CyP4. In contrast, co-administration of E2 and CoP4 induced a pattern highly resembling OVX. Bioinformatics analyses further revealed clear disparities in functional profiles associated with E2+CoP4 and E2+CyP4. Genes involved in mitochondrial energy (ATP synthase α subunit; Atp5a1), redox homeostasis (peroxiredoxin 5; Prdx5), insulin signaling (insulin-like growth factor I; Igf1), and cholesterol trafficking (liver X receptor α subtype; Nr1h3), differed in direction of regulation by E2+CoP4 (down-regulation relative to OVX) and E2+CyP4 (up-regulation relative to OVX). In contrast, genes involved in amyloid metabolism (β-secretase; Bace1) differed only in degree of regulation, as both E2+CoP4 and E2+CyP4 induced down-regulation at different efficacy. E2+CyP4-induced changes could be associated with regulation of progesterone receptor membrane component 1(Pgrmc1). In summary, results from this study provide evidence at the molecular level that differing regimens of hormone therapy (HT) can induce disparate gene expression profiles in brain. From a translational perspective, confirmation of these results in a model of natural menopause, would imply that the common regimen of continuous combined HT may have adverse consequences whereas a cyclic combined regimen, which is more physiological, could be an effective strategy to maintain neurological health and function throughout menopausal aging.

## Introduction

Combinations of estrogens and progestogens in varying regimens are used world-wide as hormone therapy (HT) for menopause-related climacteric symptoms [1], [2]. Despite the widespread use of HT, the neurological impact of clinically relevant chronically administered HT combinations and regimens is less understood than associations with cancer and vascular disease.

Preclinical and clinical data indicate that the composition of HT, in particular the progestogen component, is a critical factor in the impact of HT on cognition [3]. The largest clinical trial of HT for neural outcomes, the Women's Health Initiative Memory Study (WHIMS), indicated an adverse effect of combined HT of conjugated equine estrogens (CEE) and medroxyprogesterone acetate (MPA) on neurological health when initiated in women aged 65 years and older [4], [5]. In contrast, neither benefit nor detriment was observed among women treated with CEE alone [6], [7]. The different outcomes between CEE+MPA and CEE alone trials suggest that the addition of the synthetic progestogen, MPA, to the estrogen formulation exacerbated neurological decline in some postmenopausal women 70 years or older. These clinical findings are consistent with preclinical observations from animal models demonstrating that MPA antagonized the positive effects of 17β-estradiol (E2) on neuronal mitochondrial function and survival and synaptic plasticity [2], [8].

The principle endogenous progestogen, progesterone (P4), has both direct effects on the female brain including the hippocampus and cortex, and indirect effects through interactions with estrogen [3]. Our preclinical studies indicated that synergistic versus antagonistic interactions of P4 with E2 are sensitive to specific treatment paradigms. In both *in vitro* and *in vivo* studies, the acute simultaneous exposure to the combination of E2 and P4 antagonized the benefits of exposure to E2 alone [9], [10]. In a transgenic mouse model of Alzheimer's disease (AD) treated with E2 and P4 for 3 months, the β-amyloid (Aβ)-reducing action of E2 was blocked by continuous exposure to P4 for 90 days, while cyclic exposure to P4 for 3×10 day periods significantly reduced Aβ levels when used alone and enhanced E2-induced Aβ reduction [11], [12]. Conversely, in the hypothalamic regulation of ovarian cycles, continuous P4 protected mice from hypothalamic desensitization induced by continuous exposure to E2 [13]. Collectively, these data suggest that continuous versus cyclic regimens of HT have substantially divergent impact on brain outcomes critical to optimal neural function and prevention of AD and other neurodegenerative diseases. Another complexity is that rodent reproductive cycles (4–5 days) and pregnancy (18–20 days) are short relative to the duration of human HT (typically, 30–90 day phases), which few studies have addressed at the level of gene expression.

The present study determined, at the gene expression level, the differential impact of continuous versus cyclic exposure to P4 alone or in combination of E2, on the hippocampus, a brain region involved in cognition that is damaged in AD. Adult female rats were ovariectomized (OVX) and given continuous E2 replacement with two P4 schedules, continuous P4 (CoP4) or cyclic P4 (CyP4). The hippocampal expression of a focused group of genes involved in pathways/processes including bioenergetics, metabolism and inflammation that have been widely associated with cognitive aging and AD [14], [15], [16], [17], were profiled by qRT-PCR. A bioinformatics analysis further determined gene networks, signaling and metabolic pathways, as well as biological functions and diseases that can be potentially perturbed by different regimens. These analyses indicate profound differences between continuous versus cyclic exposure to P4 on genomic and associated functional regulation indicative of neurological heath or vulnerability to brain metabolic deficits.

## Results

**Figure 1:**
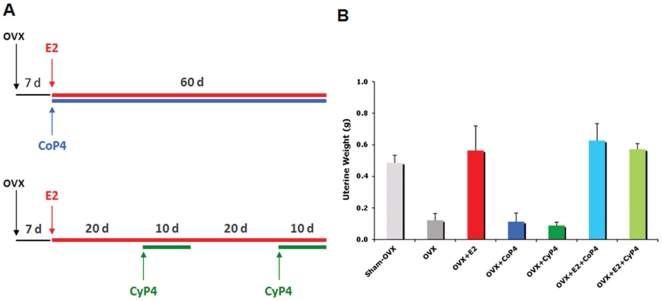
Hormone interventions and treatment paradigms (A) and uterine responses (B). OVX: ovariectomy; E2: 17β-estradiol; CoP4: continuous P4; CyP4: cyclic P4.

Seven intervention groups, Sham-OVX (treated with vehicle only), OVX (treated with vehicle only), OVX treated with E2 alone (OVX+E2), OVX treated with continuous P4 (OVX+CoP4), OVX treated with cyclic P4 (OVX+CyP4), OVX treated with E2 in combination with continuous P4 (OVX+E2+CoP4), and OVX treated with E2 in combination with cyclic P4 (OVX+E2+CyP4), were included in the present study (Fig. 1A). Endocrine responses to different hormone interventions and treatment paradigms were confirmed by uterine weight (Fig. 1B). Consistent with previous reports, when compared to Sham-OVX, hormone deprivation induced by OVX led to an approximate 50% reduction in uterine weight (P<0.01). Uterine weight in E2 treatment groups including OVX+E2, OVX+E2+CoP4 and OVX+E2+CyP4 did not differ from Sham-OVX. Uterine weight in P4 alone treatment groups including OVX+CoP4 and OVX+CyP4 did not differ from OVX (Fig. 1B).

Hippocampal gene expression in response to different hormone interventions and treatment paradigms were comparatively analyzed using qRT-PCR-based Taqman low-density arrays (TLDAs). Selection of control genes for data normalization was based upon the control stability measure (*M*). Among 4 candidate control genes, Gapdh exhibited the most stable expression across all samples, with the lowest *M* = 0.52 (as compared to Actb (*M* = 0.54), Hprt1 (*M* = 0.76) and 18 S (*M* = 0.82)), and therefore was used as the control gene in the following data analyses. Of the 163 target genes assayed, 24 genes (listed in grey in Table S1), were undetermined for at least one of two reasons: 1) no amplification possibly due to low expression or failed assays; 2) two or fewer samples with undetectable Ct values within any of the seven groups. A total of 139 genes (Ct values listed in Table S2) were analyzed for their fold change in expression relative to the OVX comparison group. Among them, 49 genes exhibited a fold change with P<0.05 relative to OVX in at least one of the 6 treatment groups (Table S3).

### CoP4 and CyP4 differentially regulate gene expression profiles

#### Mitochondrial energy and redox metabolism

**Figure 2:**
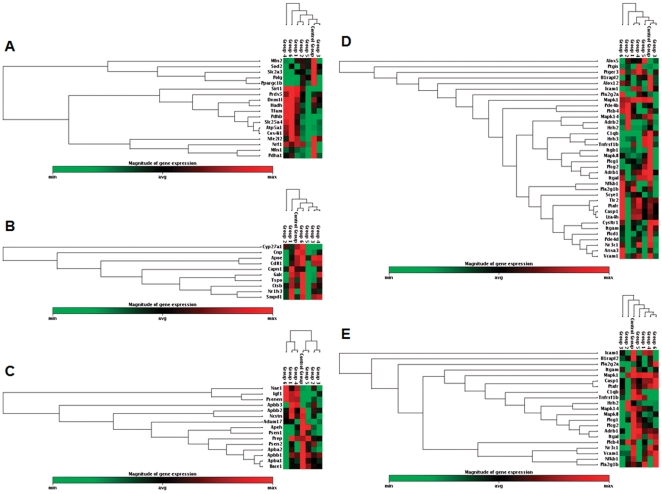
Hierarchical cluster diagrams: (A) mitochondrial energy/redox metabolism related genes (18 genes; P<0.1); (B) cholesterol homeostasis/myelin metabolism related genes (10 genes; P<0.1); (C) insulin signaling/amyloid metabolism related genes (15 genes; P<0.1); (D) inflammation related genes (36 genes; P<0.1); (E) inflammation related genes (21 genes; P<0.05). Red indicates high-expressing genes, green indicates low-expressing genes. Control Group = OVX; Group 1 = Sham-OVX; Group 2 = OVX+E2; Group 3 = OVX+CoP4; Group 4 = OVX+CyP4; Group 5 = OVX+E2+CoP4; Group 6 = OVX+E2+CyP4; OVX: ovariectomy; E2: 17β-estradiol; CoP4: continuous P4; CyP4: cyclic P4.

Of the 24 mitochondrial energy and redox metabolism related genes analyzed, 13 genes (Atp5a1, Dnm1l, Hadh, Mfn1, Mfn2, Pdha1, Polg, Ppargc1b, Prdx5, Sirt1, Slc25a4, Slc2a3 and Sod2) exhibited a statistically significant change with P<0.05 (Table S3). Compared to OVX, major changes exhibited in Sham-OVX samples included significant up-expression of Atp5a1 (FC = 1.46; P = 0.044) and Prdx5 (FC = 1.28; P = 0.047), and significant down-expression of Polg (FC = 0.63; P = 0.012) and Sod2 (FC = 0.82; P = 0.028). The OVX-induced deficit in expression of Atp5a1 was reversed by CyP4 (FC = 1.58; P = 0.020; relative to OVX) and E2+CyP4 (FC = 1.91; P = 0.046; relative to OVX). OVX-induced deficit in expression of Prdx5 was reversed by E2+CyP4 only (FC = 1.34; P = 0.009; relative to OVX), and exacerbated by E2+CoP4 (FC = 0.76; P = 0.043; relative to OVX). Compared to OVX, E2 alone did not induce a significant change in expression of any of these genes. CoP4 and E2+CoP4 induced down-expression of select genes and no up-expressed genes relative to OVX were detected (Table S3). Hierarchal clustering diagrams illustrate the high degree of similarities in the overall gene expression patterns among E2, CoP4 and E2+CoP4 treatment groups, and among Sham-OVX, CyP4 and E2+CyP4 treatment groups (Fig. 2A).

#### Cholesterol homeostasis and myelin metabolism

Of the 17 cholesterol homeostasis and myelin metabolism related genes analyzed, 6 genes (Apoe, Cd81, Ctsb, Cyp27a1, Galc and Nr1h3) exhibited a statistically significant change with P<0.05 (Table S3). Compared to OVX, the only significant change exhibited in Sham-OVX samples was the up-expression of Nr1h3 (FC = 1.98; P = 0.031). OVX-induced deficit in the expression of Nr1h3 was reversed by E2+CyP4 only (FC = 2.07; P = 0.035; relative to OVX) (Table S3). The hierarchal clustering diagram revealed again the overall similarities among E2, CoP4 and E2+CoP4 treatment groups, and among Sham-OVX, CyP4 and E2+CyP4 treatment groups; in particular, there was a high degree of similarity among Sham-OVX and E2+CyP4 treatment groups (Fig. 2B).

#### Insulin signaling and amyloid metabolism

Of the 29 insulin signaling and amyloid metabolism related genes analyzed, 7 genes (Apba1, Apba2, Apbb1, Bace1, Igf1, Ncstn and Psen2) exhibited a statistically significant change with P<0.05 (Table S3). Compared to OVX, major changes exhibited in Sham-OVX samples included nearly significant up-expression of Igf1 (FC = 1.76; P = 0.053), and significant down-expression of Bace1 (FC = 0.77; P = 0.033). OVX-induced deficit in expression of Igf1 was reversed by CyP4 (FC = 1.83; P = 0.019; relative to OVX) and E2+CyP4 (FC = 1.95; P = 0.008; relative to OVX). Similarly, OVX-induced increase in expression of Bace1 was reversed by CyP4 (FC = 0.77; P = 0.031; relative to OVX) and E2+CyP4 (FC = 0.57; P = 0.0009; relative to OVX). In agreement with the first two panels, the clustergram demonstrated similarities in expression profiles among E2, CoP4 and E2+CoP4 treatment groups, and among Sham-OVX, CyP4 and E2+CyP4 treatment groups (Fig. 2C).

#### Inflammation

Of the 60 inflammation related genes analyzed, 21 genes (Adrb1, C1qb, Casp1, Hrh2, Icam1, Il1rapl2, Itgal, Itgam, Mapk1, Mapk14, Mapk8, Nfkb1, Nr3c1, Pla2g1b, Pla2g2a, Plcb4, Plcg1, Plcg2, Ptafr, Tnfrsf1b and Vcam1) exhibited a statistically significant change with P<0.05 (Table S3). In this panel, all significant changes relative to OVX were in the direction of down-expression, and there were no clear similarities in gene expression profiles among groups. One interesting observation was that CoP4 showed a particular impact on the expression of genes within the Mapk family, with all three genes analyzed, Mapk1, Mapk14 and Mapk8 being significantly down-regulated relative to OVX. Consistent with the gene expression data, the clustergram revealed a similar degree of divergence among groups, which, nevertheless, revealed some degree of association among Sham-OVX, CyP4 and E2+CyP4 treatment groups (Fig. 2D). This association was further strengthened when only genes that had a significant change with P<0.05 were included in the cluster analysis (Fig. 2E). Moreover, the clustergrams revealed a clear similarity between E2 and CoP4 treatment groups, and an association between OVX and E2+CoP4 treatment groups (Fig. 2E).

#### Estrogen and progesterone receptors

Of the 9 estrogen and progesterone receptor genes, only Pgr and Pgrmc1 exhibited a statistically significant change with P<0.05 (Table S3). Compared to OVX, Pgr was significantly down-regulated by E2 (FC = 0.75; P = 0.009), CoP4 (FC = 0.76; P = 0.011) and E2+CoP4 (FC = 0.73; P = 0.005). Pgrmc1 was significantly up-regulated by CyP4 (FC = 1.26; P = 0.028; relative to OVX) and E2+CyP4 (FC = 1.49; P = 0.037; relative to OVX) (Table S3).

**Figure 3:**
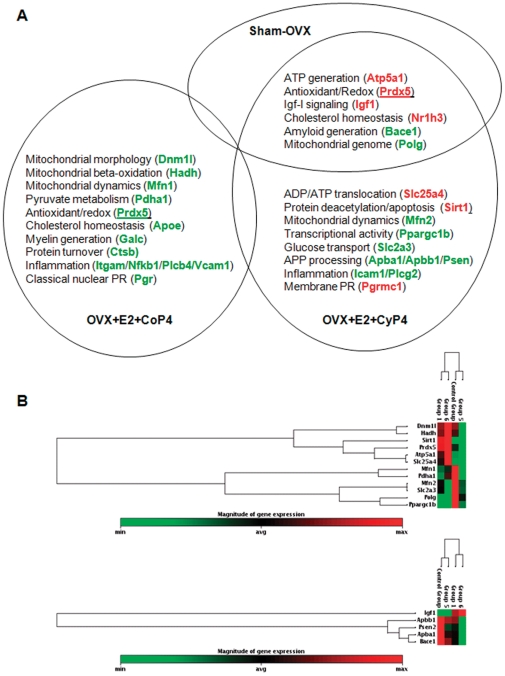
Gene expression profile associated with OVX+E2+CoP4 versus OVX+E2+CyP4 treatment paradigms. (A) Genes significantly regulated by OVX+E2+CoP4 (17 genes; P<0.05) and/or OVX+E2+CyP4 (13 genes; P<0.05); red indicates up-regulated genes, green indicates down-regulated genes. (B) Hierarchical cluster diagrams: mitochondrial energy/redox metabolism related genes (top panel; 12 genes; P<0.05); insulin signaling/amyloid metabolism related genes (lower panel; 5 genes; P<0.05). Control Group = OVX; Group 1 = Sham-OVX; Group 5 = OVX+E2+CoP4; Group 6 = OVX+E2+CyP4. OVX: ovariectomy; E2: 17β-estradiol; CoP4: continuous P4; CyP4: cyclic P4.

Taken together, data across all five focused gene panels consistently demonstrated similarities as well as divergences in the overall gene expression profiles. Substantial differences were observed between CoP4 and CyP4, particularly when co-administered with E2. Of the 17 genes significantly regulated by E2+CyP4, 7 were up-regulated and 10 were down-regulated (Fig. 3). In contrast, 13 genes significantly regulated by E2+CoP4 were all down-regulation (Fig. 3). The major divergence between E2+CoP4 and E2+CyP4 appeared to lie in their opposite effect on select genes involved in mitochondrial energy production, redox metabolism, cholesterol homeostasis, insulin signaling, and progesterone receptors (Fig. 3). By comparison, these two E2+P4 schedules appeared to have a similar down-regulatory impact on amyloid metabolism and inflammatory pathways at different efficacy or involving different genes. Moreover, E2+CyP4 share a large degree of overlap with Sham-OVX, with 6 genes similarly regulated under both conditions. In contrast, no overlap between E2+CoP4 and Sham-OVX was detected, and in fact, clustering of genes at P<0.1 or P<0.05 revealed some degree of resemblance between E2+CoP4 and OVX (Fig. 2 & 3).

### CoP4 and CyP4 differentially regulate gene functional profiles

To further understand the biological relevance of the gene expression profiles, we conducted bioinformatics analyses using IPA core application on 49 genes that exhibited significant changes (P<0.05) to determine the molecular networks, canonical pathways and biological processes (functions and diseases) that are most significantly associated with these genes.

#### Molecular networks

**Figure 4:**
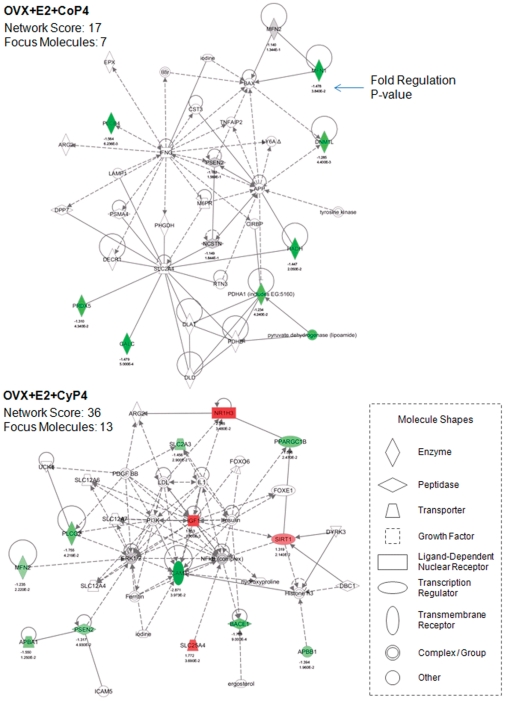
Primary molecular networks associated with OVX+E2+CoP4 versus OVX+E2+CyP4 treatment paradigms. Molecules in the network are displayed as various shapes, which indicate the molecular class. Focus molecules (colored molecules) refer to genes from the dataset that were significantly up- or down-regulated by a given treatment (OVX+E2+CoP4 or OVX+E2+CyP4); red indicates up-regulated genes (P<0.05), green indicates down-regulated genes (P<0.05), gray indicates insignificantly changed genes from the dataset (P>0.05), and white indicates molecules added from the Ingenuity Knowledge Base; color intensity indicates the degree of up or down-regulation. Lines connecting molecules indicate molecular relationships; solid lines indicate direct interaction, dashed lines indicate indirect interactions; the type of arrows indicate specific molecular relationships and the directionality of the interaction. OVX: ovariectomy; E2: 17β-estradiol; CoP4: continuous P4; CyP4: cyclic P4.

The molecular network analysis revealed how the molecules in the dataset are known to interact with one another and additional molecules in the Ingenuity Knowledge Base. Consistent with findings from the gene expression analyses, E2+CoP4 and E2+CyP4 were associated with disparate networks (Fig. 4). The primary network identified with the most relevance to E2+CoP4, with a network score of 17, includes 7 focus molecules (Dnm1l, Galc, Hadh, Mfn1, Pdha1, Plcb4, Prdx5; all of these genes were significantly regulated by E2+CoP4), and is functionally connected to cellular compromise, changes in cell morphology and neurological disease. The primary network identified with the most relevance to E2+CyP4, with a network score of 36, includes a different set of 13 focus molecules (Apba1, Apbb1, Bace1, Icam1, Igf1, Mfn2, Nr1h3, Plcg2, Ppargc1b, Psen2, Sirt1, Slc25a4, Slc2a3; all of these genes were significantly regulated by E2+CyP4), and is functionally linked to gene expression, genetic disorder and also neurological disease (Fig. 4).

**Figure 5:**
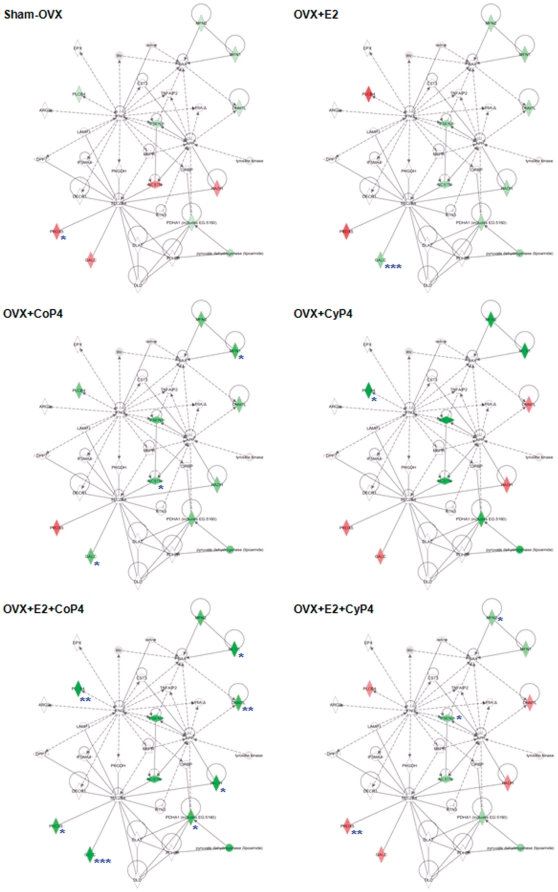
Mapping of the primary molecular network associated with OVX+E2+CoP4 with other hormone interventions and treatment paradigms. Refer to Figure 4 for the network representation, except: red indicates up-regulated genes, green indicates down-regulated genes; genes that exhibited significant changes are indicated: * P<0.05, ** P<0.01 and *** P<0.001. OVX: ovariectomy; E2: 17β-estradiol; CoP4: continuous P4; CyP4: cyclic P4.

**Figure 6:**
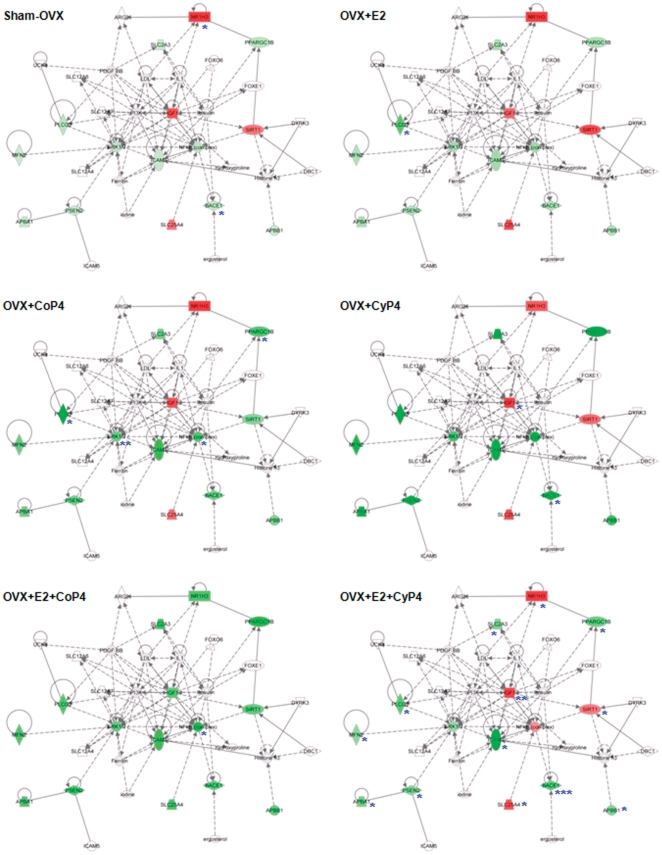
Mapping of the primary molecular network associated with OVX+E2+CyP4 with other hormone interventions and treatment paradigms. Refer to Figure 4 for the network representation, except: red indicates up-regulated genes, green indicates down-regulated genes; genes that exhibited significant changes are indicated: * P<0.05, ** P<0.01 and *** P<0.001. OVX: ovariectomy; E2: 17β-estradiol; CoP4: continuous P4; CyP4: cyclic P4.

Mapping of the primary networks associated with E2+CoP4 and E2+CyP4 with the expression data from the other five treatment groups revealed again the clear differences between E2+CoP4 and the other groups, in particular, E2+CyP4 (Fig. 5 & 6). Within the E2+CoP4-related primary network, Prdx5 appears the most significant molecule that differentiates E2+CoP4 from the remaining groups (Fig. 5). Other less significant differentiating molecules include Galc and Hadh (Fig. 5). Within the E2+CyP4-related primary network, Igf1 appears to act as a central node that differentiates E2+CoP4 from the remaining groups (Fig. 6). Other differentiators include Nr1h3, Slc25a4 and Sirt1 (Fig. 6). Molecules associated with amyloid metabolism including Apba1, Apbb1, Bace1 and Psen2, were similarly regulated by all 6 groups; Bace1 exhibited a statistically significant down-expression relative to OVX in response to Sham-OVX, CyP4 and E2+Cy4 (Fig. 6).

#### Canonical pathways

**Figure 7:**
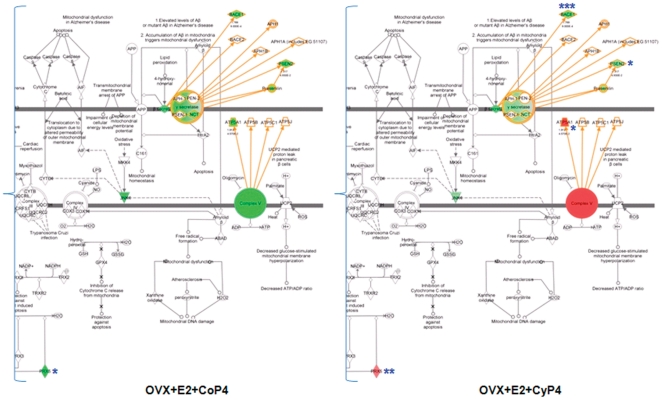
Differential impact on mitochondrial functional pathway by different hormone interventions and treatment paradigms. Molecules in the pathway are displayed as various shapes, which indicate the molecular class (refer to Figure 4). Colored molecules refer to genes from the dataset; red indicates up-regulated genes, green indicates down-regulated genes; genes that exhibited significant changes are indicated: * P<0.05, ** P<0.01 and *** P<0.001. OVX: ovariectomy; E2: 17β-estradiol; CoP4: continuous P4; CyP4: cyclic P4.

The canonical pathway analysis identified well-characterized cell signaling and metabolic pathways most relevant to the dataset. The top five most significant pathways associated with E2+CoP4 include (in order of statistical significance): LXR/RXR activation, IL-8 signaling, lymphotoxin β receptor signaling, glucocorticoid receptor signaling, and butanoate metabolism. The top five pathways associated with E2+CyP4 include (in order of statistical significance): mitochondrial function, amyloid processing, growth hormone signaling, TREM1 signaling, and glioma signaling. Mapping of the E2+CyP4-related mitochondrial pathway with the expression data from all six treatment groups reveals similar patterns to those suggested by networks. Four molecules from the gene expression dataset are included in the pathway, with Atp5a1 identified as a member of the Complex V signaling pathway, Bace1 and Psen2 as members of the APP signaling pathway, and Prdx5 as a member of the redox signaling pathway (Fig. 7). The mitochondrial energy and redox metabolism related genes, Atp5a1 and Prdx5, appear to be the key differentiators. In particular, Sham-OVX and E2+CyP4 significantly increased, whereas E2+CoP4 suppressed, Atp5a1 and Prdx5, compared to OVX. The other two amyloid metabolism-related genes, Bace1 and Psen2, were similarly down-expressed across all 6 groups relative to OVX. For clarity of display, only a comparison between E2+CoP4 and E2+CyP4 groupsis presented (Fig. 7).

#### Functions and diseases

The functional analysis enabled association of the dataset with three primary categories of biological functions: molecular and cellular functions, physiological system development and function, and diseases and disorders. Consistent with the network and pathway profiles, E2+CoP4 and E2+CyP4 were linked to different biological functions at both functional sub-categories and specific molecules involved (Table 1). At the molecular and cellular level, the top two functions associated with E2+CoP4 include cellular assembly and organization, and cell morphology. In contrast, for E2+CyP4, the top two molecular and cellular functions are gene expression and cell survival (Table 1). At the physiological system level, both E2+CoP4 and E2+CyP4 are associated with nervous system development and function; however, the specific molecules involved differ. E2+CoP4 shows a greater relevance to tissue development, whereas E2+CyP4 is more relevant to behavior such as learning and memory (Table 1). At the diseases and disorders level, while both E2+CoP4 and E2+CyP4 are highly associated with neurological disease such as dementia and more specifically Alzheimer's disease, distinct processes and molecules are regulated by these two regimens. E2+CoP4 is linked to processes related to cholesterol metabolism and myelin generation (Apoe, Ctsb, Galc, Hadh), inflammation (Nfkb1, Plcb4, Vcam1), and Pgr. Whereas E2+CyP4 is linked to processes related to mitochondrial energy and redox metabolism (Atp5a1, Mfn2, Polg, Ppargc1b, Sirt1, Slc25a4, Slc2a3), insulin signaling and amyloid metabolism (Apba1, Apbb1, Bace1, Igf1, Psen2), and Pgrmc1 (Table 1).

**Table 1:** Top functions/diseases associated with OVX+E2+CoP4 versus OVX+E2+CyP4 treatment paradigm.

Functional Category	OVX+E2+CoP4	OVX+E2+CyP4
Molecular and Cellular Function	Cellular Assembly and Organization• 7 molecules: Apoe, Ctsb, Dnm1l, Mfn1, Nfkb1, Pgr, Vcam1• P-value range: 1.34E-06–8.70E-03• Relevant functions include (in the order of statistical significance): density; distribution; quantity; morphology; formation; nucleation; disruption; export; interconnectivity; rearrangement; tubulation; outgrowth; accumulation; leakage; volume; fusion; reorganization; biogenesis; localization; proliferation; aggregation	Gene Expression• 7 molecules: Apba1, Apba2, Igf1, Nr1h3, Polg, Ppargc1b, Sirt1• P-value = 3.68E-06–6.83E-03• Relevant functions include (in the order of statistical significance): co-activation; activation; transactivation; transcription; binding
	Cell Morphology• 7 molecules: Apoe, Dnm1l, Itgam, Mfn1, Nfkb1, Pgr, Vcam1• P-value = 2.11E-06–9.00E-03• Relevant functions include (in the order of statistical significance): length; cell spreading; morphology; shape change; cellularity; innervation; tubulation; outgrowth; volume; frequency; size	Cell Survival• 12 molecules: Apbb1, Bace1, Icam1, Igf1, Mfn2, Nr1h3, Plcg2, Prdx5, Psen2, Sirt1, Slc25a4, Slc2a3• P-value = 5.61E-06–6.65E-03• Relevant functions include (in the order of statistical significance): cell death; apoptosis; survival; loss; necrosis; regeneration; cytotoxicity; activation-induced cell death; anoikis; cell viability
Physiological System Development and Function	Nervous System Development and Function• 8 molecules: Apoe, Dnm1l, Itgam, Mfn1, Nfkb1, Plcb4, Pgr, Vcam1• P-value = 1.34E-05–8.70E-03• Relevant functions include (in the order of statistical significance): density; cell viability; distribution; morphology; innervation; quantity; outgrowth; differentiation; long-term depression; long-term memory; length	Nervous System Development and Function• 8 molecules: Apbb1, Bace1, Icam1, Igf1, Mfn2, Nr1h3, Psen2, Sirt1• P-value = 4.40E-05–5.70E-03• Relevant functions include (in the order of statistical significance): Neurogenesis; proliferation; memory; survival; density; formation; spatial learning; spatial memory; neurological process; neuroprotection; cell movement; growth; quantity; size; stimulation; vascularization; accumulation; activation; branching; height; inhibition; shrinkage; stabilization; volume; area; morphology; myelination; plasticity; regeneration; innervation
	Tissue Development• 5 molecules: Apoe, Itgam, Nfkb1, Pgr, Vcam1• P-value = 7.03E-06–8.70E-03• Relevant functions include (in the order of statistical significance): adhesion; accumulation; development; branching; binding; formation; generation; adherence; cell-cell contact; growth; aggregation	Behavior• 6 molecules: Apba1, Apbb1, Bace1, Igf1, Ppargc1b, Psen2• P-value = 1.67E-05–4.56E-03• Relevant functions include (in the order of statistical significance): learning; spatial learning; hyperactivity; memory; spatial memory; anxiety; mating behavior; locomotion
Diseases	Neurological Disease• 11 molecules: Apoe, Ctsb, Dnm1l, Galc, Hadh, Mfn1, Nfkb1, Pdha1, Pgr, Plcb4, Vcam1• P-value = 4.07E-05–6.97E-03• Relevant diseases include (in the order of statistical significance): neurological disorder; neurodegenerative disorder; dementia; astrocytosis; cerebrotendinous xanthomatosis; microgliosis; Alzheimer's disease; disaggregation; age-related hearing loss; atrophy; enlargement; Krabbe's disease; stroke; organismal abnormalities; tumorigenesis	Neurological Disease• 15 molecules: Apba1, Apbb1, Atp5a1, Bace1, Icam1, Igf1, Mfn2, Pgrmc1, Plcg2, Polg, Ppargc1b, Psen2, Sirt1, Slc25a4, Slc2a3• P-value = 2.89E-08–4.56E-03• Relevant diseases include (in the order of statistical significance): dementia; Alzheimer's disease; neurological disorder; familial Alzheimer's disease; retinopathy; gliosis; infarction; Charcot-Marie-Tooth disease type 2A1; amyotrophic lateral sclerosis

## Discussion

Using qRT-PCR-based Taqman low-density gene arrays and IPA-based bioinformatics, we investigated the bioenergetics, metabolism and inflammation-related genomic and associated functional impact of ovarian hormonal depletion induced by OVX and exposure to E2/CoP4/CyP4 in the adult female rat hippocampus. Compared to Sham-OVX, OVX induced a number of significant gene expression changes, which were to a large extent reversed by co-administration of E2 and cyclic P4 (E2+CyP4). Co-administration of E2 and continuous P4 (E2+CoP4) induced a markedly disparate profile from that induced by E2+CyP4; the E2+CoP4 gene expression profile exhibited some degree of resemblance to the OVX profile.

OVX-induced changes differentiating E2+CoP4 and E2+CyP4 included genes involved in mitochondrial energy and redox metabolism, with major genes including Atp5a1 and Prdx5, respectively. Atp5a1 encodes the α subunit of mitochondrial ATP synthase catalyzing ADP to ATP production [18]. ATP synthase, the complex V which is the last complex of the mitochondrial respiratory chain, plays a central role in synthesis of the majority of ATP demanded in aerobic organs, particularly in metabolically active organs such as brain [19]. Regulation of ATP synthase is involved in physiological processes underlying normal nervous system functions, as well as neurodegenerative processes/diseases such as AD [20], [21]. In a recent study, APP and Aβ bound to ATP synthase α subunit and regulated its activity at the surface of neural cells, suggesting a novel mechanism in Aβ-mediated AD pathology [22]. The ATP synthase α subunit also is particularly vulnerable to oxidative damage compared to other components of the mitochondrial respiratory chain at earlier AD stages, suggesting that the oxidation of ATP synthase and alterations of its biological functions may be an early and essential step in the pathogenesis of AD [23].

Peroxiredoxins (Prxs) are a family of thiol-dependent antioxidant enzymes that catalyze the reduction of peroxides [24], [25]. Different from conventional antioxidants, Prxs exhibit unique properties including high enzymatic activity through utilization of cysteine at their active site and high cellular abundance and involvement in both antioxidant defense and redox signaling [24], [25]. Six Prx isozymes have been identified in mammalian cells sharing a common peroxide-scavenging activity despite differences in their cellular distribution and subcellular localization. Prx1 and Prx6 are expressed in astrocytes and glial cells; Prx2, Prx3, Prx4, and Prx5 (encoded by Prdx5) are expressed in neurons [26]. Subcellularly, Prx1, Prx2 and Prx6 are mainly localized in the cytoplasm, Prx4 in the endoplasmic reticulum, Prx3 in the mitochondria; Prx5 is expressed in various compartments including peroxisomes and mitochondria [26]. Since mitochondria are a major site of generation of and target for hydrogen peroxide, the mitochondrial Prxs, including Prx3 and Prx5, play an important role in regulation of mitochondrial redox homoeostasis [27]. Prx5 in particular may present broader activity than other antioxidant mitochondrial enzymes [28], [29]. Overexpression of Prx5 has a positive impact on neuronal survival and extension of lifespan; Prx5 deficiency produces signs of oxidative stress, apoptosis, and shortened lifespan [26], [27].

Our findings of deficits in the expression of genes involved in mitochondrial energy and redox metabolism in the hippocampus of OVX rat brain are consistent with our previous observations indicating vulnerability of CNS bioenergetic processes to female endocrine aging. For instance, in female mice, we discovered a close link between reproductive senescence and neuronal bioenergetic deficit demonstrated by a significant decline in complex IV cytochrome c oxidase activity and mitochondrial respiration [30]. The vulnerability of neuronal bioenergetics appeared to be more prominent in AD brain, indicated by our finding in 3xTg-AD mice that mitochondrial bioenergetic deficits in the brain preceded the development of AD pathology [16]. Results from the present study emphasize compromised mitochondrial energy and redox metabolism with ovarian disruption and are consistent with earlier reports of age and AD related deficits in bioenergetics [14], [15], [17], [31]. A regimen of E2 combined with a cyclic, not continuous, P4, could be an effective strategy for rescuing brain from hormonal deficiency-related brain hypometabolism and increased oxidative stress.

Another major change induced by OVX and differentially regulated by E2+CoP4 and E2+CyP4 was in expression of Igf1. On bioinformatics analysis, Igf1 appeared to act as a hub in the most significant network associated with E2+CyP4. Igf1 encodes the insulin-like growth factor I (Igf-I) that serves as a master regulator of a wide spectrum of biological functions and homeostasis in multiple tissues [32]. Igf-I plays an essential role in the modulation of neural activities from early development to adult neurogenesis, to cognition, and to protection against neurodegenerative diseases [33]. Igf-I modulates brain levels of Aβ via regulation of proteins involved in major pathways leading to catabolic removal of Aβ [34], [35], [36]. Igf-I is also regulated by a number of other factors, including the sex steroids E2 and P4. Substantial evidence indicates crosstalk between E2 and Igf-I signaling pathways to regulate multiple neural responses including cognition [37], [38]. Relative to E2, the impact of P4 on the Igf-I system has been less explored. A recent report suggests that P4 may positively regulate the Igf-I system in glial cells, which could be directly linked to a P4-mediated protection against demyelination [39]. Data from the present study supports the close interactions between Igf-I and E2/P4; Igf-I deficit associated with hormonal deficiency could be effectively reversed by chronic exposure to E2+CyP4.

Additional changes induced by OVX and differentially regulated by E2+CoP4 and E2+CyP4 included genes involved in cholesterol homeostasis and APP intramembranous processing, major players including Nr1h3 and Bace 1 respectively. Nr1h3 encodes the α subtype of the liver X receptor (Lxrα), which, along with Lxrβ (Nr1h2), are oxysterol receptors that function as master transcription factors mediating cholesterol homeostasis in the periphery [40]. In the brain, emerging evidence indicates that, in addition to the regulation of cholesterol transport and metabolism, activation of Lxrs also attenuates inflammatory responses and Aβ production, supporting their therapeutic potential in AD management and other neurological disorders involving dysregulation of cholesterol homeostasis [41]. Bace1 encodes β-secretase, the β-site APP-cleaving enzyme I. [42]. Since Bace1 cleavage of APP is a pre-requisite for Aβ generation, and the level and activity of Bace1 are elevated in AD, Bace1 has been suggested as a biological candidate marker for early detection of AD [43]. Data from the present study suggest that loss of ovarian hormones may increase risk for the development of AD through deficits in cholesterol and APP metabolism, which may be prevented by chronic exposure to E2+CyP4. Compared to pathways discussed above that were regulated in different direction by E2+CoP4 versus E2+CyP4, the APP processing pathway appeared to be a less dramatic differentiator since the key genes (Bace1, Psenen) involved in the pathway were repressed by both E2+CoP4 and E2+CyP4.

The disparity between E2+CoP4 and E2+CyP4 was also reflected on their differential regulation of P4 receptors, Pgr versus Pgrmc1. Pgr is the classical nuclear PR and has been associated with many neural responses to P4 [3]. In contrast to most reports utilizing acute HT, we found that chronic HT treatment paradigms used in the present study including E2, CoP4 and E2+CoP4 decreased Pgr. In chick embryos, acute P4 exposure increased Pgr in hypothalamus and posterior pituitary [44]. In adult OVX rats, E2 and combined E2 and P4 increased Pgr in several hypothalamic nuclei, while P4 alone was not different from OVX [45]. Although some studies document that P4 can antagonize E2-mediated upregulation of Pgr [46], others did not find P4 antagonism of E2 induction of Pgr [45], [47]. Recent data from our group indicate regional differences in Pgr regulation by E2 and P4 within the adult rat hippocampus [48]. Moreover, in the hypothalamus, OVX decreased Pgr by 80% in the medial preoptic nucleus but by only 20% in the ventromedial nucleus [49]. We know of only one other report of Pgr response to chronic hormone exposure [50]. Continuous E2 treatment for 8 weeks in OVX rats did not increase Pgr in the hypothalamus and hippocampus [50]. Together, these data suggests that Pgr regulation by E2 and P4 could be highly dependent upon the duration of hormone treatment, as well as brain region.

In contrast to Pgr, Pgrmc1 is a membrane-associated PR and a member of a multi-protein complex regulating a spectrum of hormone-signaling pathways [51]. Pgrmc1 is expressed in multiple brain regions, mediates P4-induced responses in multiple neural cell types, and is suggested to play a role in the promotion of neonatal dendritic growth, spino- and synapto-genesis [52], [53], [54]. Further, our previous analysis revealed that Pgrmc1 mediated P4-induced increase in cell cycle gene expression and proliferation in rat neural progenitor cells [55]. The present study demonstrated that CyP4 alone or with E2 induced an up-regulation of Pgrmc1. E2 and CyP4 interacted synergistically since the response to E2+CyP4 was greater than the response induced by CyP4 alone. In contrast, an antagonistic interaction occurred between E2 and CoP4 where Pgrmc1 expression decreased. Together, these results lead to the hypothesis that the disparities in gene expression patterns between E2+CoP4 and E2+CyP4 could be closely associated with their differential regulation of Pgrmc1.

It should be pointed out that, technically and statistically, this study presents both strengths and limitations. One issue arisen was the consideration of adjustment for multiple hypothesis testing: an approach such as consideration of the false positive discovery rate, in particular, is usually required in microarray studies where thousands of genes are analyzed simultaneously. Because of the relatively large chance for false positive discoveries involved in such high density microarray studies, findings usually need to be further validated by real-time RT-PCR analysis. In our study, we directly used the real-time RT-PCR technique following a carefully developed and validated protocol, and as part of this protocol, our low-density array consisting of a pathway-focused set of target genes was custom designed based on prior literature and hypothesized associations with neurological aging dysfunctions as well as hormone therapy interventions. Therefore, in consideration of three factors: 1) the well-designed and quality-controlled technical strengths, 2) the intent to reduce false negatives in this first-ever analysis, 3) the by and large internal agreement of our findings with published functional data, we did not control for multiple hypothesis testing, and instead, chose to present unadjusted p-values in this study. Given these reasons, we however recognize the possibility of false positive associations and the need for independent validation in larger studies.

Collectively, our findings from the present study demonstrate that, consistent with many preclinical and clinical observations, surgically-induced ovarian hormone loss exerts a negative impact on multiple biological processes in the brain, including mitochondrial energy production, antioxidant defense/redox homeostasis, cholesterol trafficking, insulin signaling and amyloid metabolism, all of which, as discussed above, play a critical role in neurological health and disease. Further, data contained herein indicate that systems of gene expression in brain are differentially impacted by exposure to cyclic versus continuous hormone interventions and treatment paradigms. A regimen of E2 combined with a cyclic exposure to P4, which more closely mimics the natural female hormone pattern, induced gene expression profiles consistent with the ovary intact brain. In stark contrast, a regimen of E2 combined with a continuous combined exposure to P4, which more closely mimics the clinical hormone therapy regimen, induced gene expression profiles consistent with the ovarian hormone deficient brain. These preclinical data require confirmation in a model of natural menopause but suggest that a hormone therapy of E2 and cyclic exposure to P4 would be a promising therapeutic strategy for preventing or reducing hormonal deficiency-induced compromise on neurological health and function.

## Materials and Methods

### Chemicals

E2/P4 and corresponding placebo pellets manufactured for long term continuous release were purchased from Innovative Research of America (Sarasota, FL).

### Animals and treatment

The use of animals and treatment were approved by the Institutional Animal Care and Use Committee at the University of Southern California (Protocol No. 10911). Three-month-old Sprague-Dawley female rats were ovariectomized (OVX) at Harlan (Indianapolis, IN). Animals were randomized into 7 treatment groups (N = 8/group): Sham-OVX (treated with vehicle only), OVX (treated with vehicle only), OVX+E2, OVX+CoP4, OVX+CyP4, OVX+E2+CoP4, OVX+E2+CyP4 (Fig. 1A). For E2 (treated with the 190 mg/60 d pellets) and CoP4 (treated with the 450 mg/60 d pellets) exposure, pellets were subcutaneously implanted on day_7 after OVX and remained for the entire 60_day experiment. For CyP4 (treated with the 50 mg/10 d pellets) exposure, pellets were subcutaneously inserted on day_20 and day_50 after E2 treatment (day_27 and day_50 after OVX). Animals were killed by perfusion on day_60 after initiation of E2/P4 treatment (day_67 after OVX) (Fig. 1A). The uterus was collected and the uterine weight was used to confirm hormone treatments (Fig. 1B). Brain hemispheres were dissected and frozen. Hippocampal tissues yielded from 4 animals per group were used for gene expression profiling analysis (tissues from the other 4 animals were used for protein and other biochemical analyses).

### TLDA gene expression profiling

Taqman low-density array (TLDA) technology, based upon the quantitative RT-PCR platform, has recently emerged as a novel tool for gene expression profiling, providing a focused, sensitive, reproducible and medium-high throughput approach to simultaneously measure expression of up to 384 genes in single samples [56].

In the present study, TLDA cards (Format 48, which enables analysis of 4 samples in duplicate against 48 different genes) were custom manufactured at Applied Biosystems (Foster City, CA), and loaded with Taqman expression assays for target genes divided into five functional groups and 4 assays for candidate control genes (Table S1). Total RNA was isolated from rat hippocampal tissues using the PureLink RNA Mini Kit (Invitrogen, Carlsbad, CA). RNA quantity and quality were analyzed using the Experion RNA StdSens Analysis Kit on an Experion Automated Electrophoresis System (Bio-Rad, Hercules, CA). The integrity of RNA samples was assessed by the Relative Quality Indicator (RQI, scale 1–10 colored from red to orange to green) [57]. RNA samples with the RQI reading greater than 8.0 were advanced to cDNA synthesis. RNA to cDNA synthesis was prepared using the High Capacity RNA-to-cDNA Master Mix (Applied Biosystems) on a MyCycler Thermal Cycler (Bio-Rad). Taqman real-time qRT-PCR reactions were performed on 50 ng cDNA samples mixed with the TaqMan Universal PCR Master Mix 2× (Applied Biosystems), under the thermal cycling conditions: stage 1: AmpErase UNG activation at 50°C/2 min/100% ramp; stage 2: AmpliTaq gold DNA polymerase activation at 94.5°C/10 min/100% ramp; stage 3: melt at 97°C/30 sec/50% ramp, followed by anneal/extend at 59.7°C/1 min/100% ramp, for 40 cycles. Fluorescence was detected on an ABI 7900HT Fast Real-Time PCR System equipped with the Sequence Detection System Software Version 2.3 (Applied Biosystems).

Data were analyzed using the RQ Manager Version 1.2 and DataAssist Version 2.0 (Applied Biosystems). Relative gene expression levels or fold changes relative to the comparison group (OVX treated with vehicle only) were calculated by the comparative Ct (ΔΔCt) method, with Ct denoting threshold cycle [58]. Selection of the endogenous control gene for normalization was based on the control stability measure (*M*), which indicates the expression stability of control genes on the basis of non-normalized expression levels. *M* was calculated using the geNorm algorithm; genes with the lowest *M* values have the most stable expression. Four samples (collected from 4 animals) per group were included in the analysis. For each sample, average Ct for each target gene was calculated as the mean of 2 technical replicates; ΔCt was calculated as the difference in average Ct of the target gene and the endogenous control gene. For each treatment group, mean 2^−ΔCt^ was calculated as the geometric mean of 2^−ΔCt^ of the 4 samples in the group. Fold change was then calculated as mean 2^−ΔCt (treatment group)^/mean 2^−ΔCt (comparison group)^. Fold change values greater than one indicate a positive expression or up-regulation relative to the comparison group. Fold change values less than one indicate a negative expression or down-regulation relative to the comparison group. Fold regulation was used to represent the fold change results in a biologically meaningful way. For fold change values greater than one (up-regulation), the fold regulation is equal to the fold change; for fold change values less than one (down-regulation), the fold regulation is the negative inverse of the fold change. The 2^−ΔCt^ values for each target gene were statistically compared between the treatment and comparison group using Student's t-test. As a result, a total of 139 target genes were analyzed for expression changes between 6 treatment groups and the OVX control group, which yielded a total of 139×6 = 834 statistical tests. The statistical significance was indicated by * P<0.05, ** P<0.01 and *** P<0.001; P-values were unadjusted by multiple testing corrections, a statistical method to correct for occurrence of false positive discoveries (an explanation is included in Discussion).

Hierarchical clustering diagrams graphically displayed clusters of treatment groups as well as target genes. Distances between treatment groups/target genes, shown in maximum linkage, were calculated by clustering algorithm Pearson's correlations based on the ΔCt values.

### IPA gene functional profiling

IPA (Ingenuity Systems, Redwood City, CA), which leverages the Ingenuity Knowledge Base, the largest database housing biological and chemical relationships extracted from the scientific literature, provided a bioinformatics computing tool to interpret our experimental gene expression dataset in the context of biological processes, pathways, and molecular networks (www.ingenuity.com). Data including only statistically significantly altered genes (P<0.05) containing gene identifiers and corresponding fold regulation values was uploaded into IPA 8.7 and analyzed by a core analysis composed of a network analysis, a canonical pathway analysis and a function/disease analysis.

The network analysis identified biological connectivity among molecules in the dataset that were significantly up- or down-regulated by a given treatment (these molecules are called “network eligible molecules” or “focus molecules” that serve as “seeds” for generating networks) and their interactions with other molecules present in the Ingenuity Knowledge Base. Network eligible molecules were combined into networks that maximized their specific connectivity. Additional molecules from the Ingenuity Knowledge Base (these molecules are called “interacting molecules”) were used to specifically connect two or more smaller networks to merge them into a larger one. A network was composed of direct (requiring direct physical contact between two molecules) and indirect (mediated by intermediate factor(s)) interactions among focus molecules and interacting molecules, with a maximum of 35 molecules per network. Generated networks were ranked by the network score according to their degree of relevance to the network eligible molecules from the dataset. The network score was calculated with Fisher's exact test, taking into account the number of network eligible molecules in the network and the size of the network, as well as the total number of network eligible molecules analyzed and the total number of molecules in the Ingenuity Knowledge Base that were included in the network. Higher network scores are associated with lower probability of finding the observed number of network eligible molecules in a given network by chance.

The canonical pathway analysis identified the cell signaling and metabolic pathways stored in the Ingenuity Knowledge Base that were most significant to the dataset. The significance of the association between the dataset and a given canonical pathway was measured in two ways: 1) a ratio of the number of molecules from the dataset that map to the pathway divided by the total number of molecules that are contained in that pathway, and 2) a P-value calculated by Fisher's exact test. P-values less than 0.05 indicate a statistically significant, non-random association between a set of molecules in the dataset and a given canonical pathway.

The functional analysis identified the biological functions and diseases that were most significant to the dataset. Fisher's exact test was used to calculate the P-value, considering 1) the number of molecules in the dataset that participate in that function/disease, and 2) the total number of molecules that are known to be associated with that function/disease in the Ingenuity Knowledge Base. P-values less than 0.05 indicate a statistically significant, non-random association between a set of molecules in the dataset and a given function/disease.

We used the IPA network/pathway to graphically depict the biological relationships between molecules. Molecules are presented as nodes, and the biological relationship between two modes is represented as an edge (line). All edges are supported by at least one reference from the literature, from a textbook, or from canonical information stored in the Ingenuity Knowledge Base. The intensity of the node color indicates the degree of up- (red) or down- (green) regulation relative to the comparison group. Nodes are displayed using various shapes that represent the functional class of the gene product. Edges are displayed with various labels that describe the nature of the relationship between the nodes. Solid lines indicate direct relationships, dashed lines indicate indirect relationships, and circular arrows or lines indicate self-referential relationships that arise from the ability of a molecule to act upon itself.

## Supporting Information

Table S1 **Taqman gene expression assays.**
(DOCX)

Table S2 **Gene expression Ct values.**
(XLS)

Table S3 **Gene expression changes in response to different hormone interventions and treatment paradigms.**
(DOCX)
