# Supplementary material for: Continuous versus Cyclic Progesterone Exposure Differentially Regulates Hippocampal Gene Expression and Functional Profiles
Source: PLoS One. 2012 Feb 29;7(2):e31267. doi: 10.1371/journal.pone.0031267 (PMC3290616; doi:10.1371/journal.pone.0031267)
Supplement: Table S1 — Taqman gene expression assays. (DOCX) [file pone.0031267.s001.docx]

**Table S1:** Taqman gene expression assays.

| Functional Group | Gene Symbol | Gene Name | Assay ID |
| --- | --- | --- | --- |
| Mitochondrial Energy & Redox Metabolism | Atp5a1 | ATP synthase, mitochondrial F1 complex, alpha subunit 1 | Atp5a1-Rn01638043_g1 |
|  | Cox4i1 | cytochrome c oxidase subunit IV isoform 1 | Cox4i1-Rn00665001_g1 |
|  | Cyb5b | Cytochrome b5 type B (outer mitochondrial membrane) | Cyb5b-Rn00577982_m1 |
|  | Dnm1l | dynamin 1-like | Dnm1l-Rn00586466_m1 |
|  | Hadh | Hydroxyacyl-Coenzyme A dehydrogenase | Hadh-Rn00589352_m1 |
|  | Mfn1 | mitofusin 1 | Mfn1-Rn00594496_m1 |
|  | Mfn2 | mitofusin 2 | Mfn2-Rn00500120_m1 |
|  | MT-CO2 | Cytochrome c oxidase II | MT-CO2-Rn03296737_s1 |
|  | MT-CO3 | Cytochrome c oxidase III | MT-CO3-Rn03296820_s1 |
|  | Nfe2l2 | nuclear factor (erythroid-derived 2)-like 2 | Nfe2l2-Rn00477784_m1 |
|  | Nrf1 | nuclear respiratory factor 1 | Nrf1-Rn01455958_m1 |
|  | Pdha1 | pyruvate dehydrogenase (lipoamide) alpha 1 | LOC685778-Rn01424350_g1 |
|  | Pdhb | pyruvate dehydrogenase (lipoamide) beta | Pdhb-Rn01537771_g1 |
|  | Polg | polymerase (DNA directed), gamma | Polg-Rn00450527_m1 |
|  | Ppargc1a | peroxisome proliferator-activated receptor gamma, coactivator 1 alpha | Ppargc1a-Rn00580241_m1 |
|  | Ppargc1b | peroxisome proliferator-activated receptor gamma, coactivator 1 beta | Ppargc1b-Rn00598552_m1 |
|  | Prdx5 | Peroxiredoxin 5 | Prdx5-Rn00586040_m1 |
|  | Sirt1 | sirtuin | Sirt1-Rn01428093_m1 |
|  | Slc16a1 | Solute carrier family 16, member 1 (monocarboxylic acid transporter 1) | Slc16a1-Rn00562332_m1 |
|  | Slc16a7 | Solute carrier family 16, member 7 (monocarboxylic acid transporter 2) | Slc16a7-Rn00568872_m1 |
|  | Slc25a4 | Solute carrier family 25 (mitochondrial carrier), member 4 | Slc25a4-Rn00821477_g1 |
|  | Slc2a1 | Solute carrier family 2 (facilitated glucose transporter), member 1 | Slc2a1-Rn01417099_m1 |
|  | Slc2a3 | Solute carrier family 2 (facilitated glucose transporter), member 3 | Slc2a3-Rn00567331_m1 |
|  | Sod2 | Superoxide dismutase 2, mitochondrial | Sod2-Rn00566942_g1 |
|  | Tfam | transcription factor A, mitochondrial | Tfam-Rn00580051_m1 |
| Cholesterol Homeostasis & Myelin Metabolism | Abca1 | ATP-binding cassette, sub-family A (ABC1), member 1 | Abca1-Rn00710172_m1 |
|  | Apoe | Apolipoprotein E | Apoe-Rn00593680_m1 |
|  | Apof | Apolipoprotein F | Apof-Rn01756260_g1 |
|  | Capn1 | calpain 1, (mu/I) large subunit | Capn1-Rn00569689_m1 |
|  | Cd81 | Cd81 molecule | Cd81-Rn00565272_m1 |
|  | Cnp | 2’3’-cyclic nucleotide 3’ phosphodiesterase; CNPase | Cnp-Rn01399463_m1 |
|  | Ctsb | cathepsin B | Ctsb-Rn00575030_m1 |
|  | Cyp27a1 | Cytochrome P450, family 27, subfamily A, polypeptide 1 | Cyp27a1-Rn00710297_m1 |
|  | Galc | galactosylceramidase | Galc-Rn01517759_m1 |
|  | Lcat | Lecithin-cholesterol acyltransferase | Lcat-Rn00500505_m1 |
|  | Mbp | myelin basic protein | Mbp-Rn00690431_m1 |
|  | Mog | myelin oligodendrocyte glycoprotein | Mog-Rn00575354_m1 |
|  | Nr1h2 | Nuclear receptor subfamily 1, group H, member 2 | Nr1h2-Rn00581178_m1 |
|  | Nr1h3 | Nuclear receptor subfamily 1, group H, member 3 | Nr1h3-Rn00581185_m1 |
|  | Nr1i2 | Nuclear receptor subfamily 1, group I, member 2 | Nr1i2-Rn00583887_m1 |
|  | Plp1 | proteolipid protein 1 | Plp1-Rn00456892_m1 |
|  | Smpd1 | sphingomyelin phosphodiesterase 1, acid lysosomal | Smpd1-Rn01506464_g1 |
|  | Star | Steroidogenic acute regulatory protein | Star-Rn00580695_m1 |
|  | Tspo | Translocator protein | Tspo-Rn00560892_m1 |
| Insulin Signaling & Amyloid Metabolism | A2m | Alpha-2-macroglobulin | A2m-Rn00560589_m1 |
|  | Ace | Angiotensin I converting enzyme (peptidyl-dipeptidase A) 1 | Ace-Rn00561094_m1 |
|  | Adam17 | ADAM metallopeptidase domain 17; TACE | Adam17-Rn00571880_m1 |
|  | Apba1 | Amyloid beta (A4) precursor protein-binding, family A, member 1 | Apba1-Rn00582341_m1 |
|  | Apba2 | Amyloid beta (A4) precursor protein-binding, family A, member 2 | Apba2-Rn00582349_m1 |
|  | Apba3 | Amyloid beta (A4) precursor protein-binding, family A, member 3 | Apbb3-Rn00588606_m1 |
|  | Apbb1 | Amyloid beta (A4) precursor protein-binding, family B, member 1 | Apbb1-Rn00589704_m1 |
|  | Apbb2 | Amyloid beta (A4) precursor protein-binding, family B, member 2 | Apbb2-Rn01413178_m1 |
|  | Apbb3 | Amyloid beta (A4) precursor protein-binding, family B, member 3 | Apbb3-Rn00588606_m1 |
|  | Apeh | N-acylaminoacyl-peptide hydrolase | Apeh-Rn00560727_m1 |
|  | Aph1a | Anterior pharynx defective 1 homolog A (C. elegans) | Aph1a-Rn01534325_g1 |
|  | App | Amyloid beta (A4) precursor protein | App-Rn00570673_m1 |
|  | Bace1 | Beta-site APP-cleaving enzyme 1 | Bace1-Rn00569988_m1 |
|  | Bace2 | Beta-site APP-cleaving enzyme 2 | Bace2-Rn01763455_m1 |
|  | Ece1 | Endothelin converting enzyme 1 | Ece1-Rn00585943_m1 |
|  | Ece2 | Endothelin converting enzyme 2 | Ece2-Rn01404095_m1 |
|  | Ide | Insulin-degrading enzyme | Ide-Rn00565839_m1 |
|  | Igf1 | Insulin-like growth factor 1 (somatomedin C) | Igf1-Rn00710306_m1 |
|  | Ins1 | Insulin | Ins1-Rn02121433_g1 |
|  | Insr | Insulin receptor | Insr-Rn01637243_m1 |
|  | Mme | Membrane metallo-endopeptidase | Mme-Rn00561572_m1 |
|  | Mmp2 | Matrix metallopeptidase 2 (gelatinase A, 72kDa gelatinase) | Mmp2-Rn01538177_m1 |
|  | Mmp3 | Matrix metallopeptidase 3 (stromelysin 1, progelatinase) | Mmp3-Rn00591740_m1 |
|  | Mmp9 | Matrix metallopeptidase 9 (gelatinase B, 92kDa gelatinase) | Mmp9-Rn00579162_m1 |
|  | Nae1 | NEDD8 activating enzyme E1 subunit 1 | Nae1-Rn00583368_m1 |
|  | Ncstn | Nicastrin | Ncstn-Rn00598037_m1 |
|  | Plg | Plasminogen | Plg-Rn00585167_m1 |
|  | Prep | Prolyl endopeptidase | Prep-Rn00580039_m1 |
|  | Psen1 | Presenilin 1 | Psen1-Rn00569763_m1 |
|  | Psen2 | Presenilin 2 (Alzheimer disease 4) | Psen2-Rn00579412_m1 |
|  | Psenen | Presenilin enhancer 2 homolog (C. elegans) | Psenen-Rn01476909_g1 |
|  | Thop1 | Thimet oligopeptidase 1 | Thop1-Rn00597140_m1 |
|  | Timp2 | TIMP metallopeptidase inhibitor 2 | Timp2-Rn00573232_m1 |
|  | Ubqln1 | Ubiquilin 1 | Ubqln1-Rn00587037_m1 |
| Inflammation | Adrb1 | Adrenergic, beta-1-, receptor | Adrb1-Rn00824536_s1 |
|  | Adrb2 | Adrenergic, beta-2-, receptor, surface | Adrb2-Rn00560650_s1 |
|  | Ager | Advanced glycosylation end product-specific receptor | Ager-Rn00584249_m1 |
|  | Alox12 | Arachidonate 12-lipoxygenase | Alox12-Rn01461082_m1 |
|  | Alox5 | Arachidonate 5-lipoxygenase | Alox5-Rn00563172_m1 |
|  | Anxa1 | Annexin A1 | Anxa1-Rn00563742_m1 |
|  | Anxa3 | Annexin A3 | Anxa3-Rn00563181_m1 |
|  | Anxa5 | Annexin A5 | Anxa5-Rn00565571_m1 |
|  | C1qb | Complement component 1, q subcomponent, B chain | C1qb-Rn00570480_m1 |
|  | C3 | Complement component 3 | C3-Rn00566466_m1 |
|  | Casp1 | Caspase 1, apoptosis-related cysteine peptidase | Casp1-Rn00562724_m1 |
|  | Cysltr1 | Cysteinyl leukotriene receptor 1 | Cysltr1-Rn00586294_s1 |
|  | Gfap | Glial fibrillary acidic protein | Gfap-Rn00566603_m1 |
|  | Hpgd | Hydroxyprostaglandin dehydrogenase 15-(NAD) | Hpgd-Rn00577775_m1 |
|  | Hrh1 | Histamine receptor H1 | Hrh1-Rn00566691_s1 |
|  | Hrh2 | Histamine receptor H2 | Hrh2-Rn00564216_s1 |
|  | Hrh3 | Histamine receptor H3 | Hrh3-Rn00585276_m1 |
|  | Icam1 | Intercellular adhesion molecule 1 | Icam1-Rn00564227_m1 |
|  | Il10 | Interleukin 10 | Il10-Rn01644839_m1 |
|  | Il13 | Interleukin 13 | Il13-Rn00587615_m1 |
|  | Il1a | Interleukin 1, alpha | Il1a-Rn00566700_m1 |
|  | Il1b | Interleukin 1, beta | Il1b-Rn00580432_m1 |
|  | Il1r1 | Interleukin 1 receptor, type I | Il1r1-Rn00565482_m1 |
|  | Il1r2 | Interleukin 1 receptor, type II | Il1r2-Rn00588589_m1 |
|  | Il1rapl2 | Interleukin 1 receptor accessory protein-like 2 | Il1rapl2-Rn01410545_m1 |
|  | Il1rl1 | Interleukin 1 receptor-like 1 | Il1rl1-Rn01640664_m1 |
|  | Il2ra | Interleukin 2 receptor, alpha | Il2ra-Rn00565865_m1 |
|  | Il2rb | Interleukin 2 receptor, beta | Il2rb-Rn00682353_m1 |
|  | Il2rg | Interleukin 2 receptor, gamma | Il2rg-Rn01752908_g1 |
|  | Il6 | Interleukin 6 (interferon, beta 2) | Il6-Rn00561420_m1 |
|  | Itgal | Integrin, alpha L (antigen CD11A) | Itgal-Rn01754645_m1 |
|  | Itgam | Integrin, alpha M (complement component 3 receptor 3) | Itgam-Rn00709342_m1 |
|  | Itgb1 | Integrin, beta 1 (antigen CD29 includes MDF2, MSK12) | Itgb1-Rn00566727_m1 |
|  | Itgb2 | Integrin, beta 2 (complement component 3 receptor 3/4) | Itgb2-Rn01427948_m1 |
|  | Lta4h | Leukotriene A4 hydrolase | Lta4h-Rn01503878_m1 |
|  | Ltc4s | Leukotriene C4 synthase | Ltc4s-Rn01497055_g1 |
|  | Mapk1 | Mitogen-activated protein kinase 1 | Mapk1-Rn00671828_m1 |
|  | Mapk14 | Mitogen-activated protein kinase 14 | Mapk14-Rn00578842_m1 |
|  | Mapk3 | Mitogen-activated protein kinase 3 | Mapk3-Rn00820922_g1 |
|  | Mapk8 | Mitogen-activated protein kinase 8 | Mapk8-Rn01453358_m1 |
|  | Nfkb1 | Nuclear factor of kappa B | Nfkb1-Rn01399583_m1 |
|  | Nr3c1 | Nuclear receptor subfamily 3, group C, member 1 | Nr3c1-Rn00561369_m1 |
|  | Pde4a | Phosphodiesterase 4A, cAMP-specific | Pde4a-Rn00565354_m1 |
|  | Pde4b | Phosphodiesterase 4B, cAMP-specific | Pde4b-Rn00566785_m1 |
|  | Pde4c | Phosphodiesterase 4C, cAMP-specific | Pde4c-Rn01754402_g1 |
|  | Pde4d | Phosphodiesterase 4D, cAMP-specific | Pde4d-Rn00566798_m1 |
|  | Pla2g10 | Phospholipase A2, group X | Pla2g10-Rn01424865_g1 |
|  | Pla2g1b | Phospholipase A2, group IB (pancreas) | Pla2g1b-Rn00580896_m1 |
|  | Pla2g2a | Phospholipase A2, group IIA (platelets, synovial fluid) | Pla2g2a-Rn00580999_m1 |
|  | Pla2g2d | Phospholipase A2, group IID | Pla2g2d-Rn01520520_m1 |
|  | Pla2g4c | Phospholipase A2, group IVC (cytosolic, calcium-independent) | LOC691813-Rn01772640_m1 |
|  | Plcb2 | Phospholipase C, beta 2 | Plcb2-Rn00585063_m1 |
|  | Plcb3 | Phospholipase C, beta 3 (phosphatidylinositol-specific) | Plcb3-Rn01453968_m1 |
|  | Plcb4 | Phospholipase C, beta 4 | Plcb4-Rn00577426_m1 |
|  | Plcd1 | Phospholipase C, delta 1 | Plcd1-Rn00690481_m1 |
|  | Plce1 | Phospholipase C, beta 2 | Plce1-Rn00587127_m1 |
|  | Plcg1 | Phospholipase C, gamma 1 | Plcg1-Rn00566108_m1 |
|  | Plcg2 | Phospholipase C, gamma 2 (phosphatidylinositol-specific) | Plcg2-Rn00567751_m1 |
|  | Ptafr | Prostaglandin F receptor (FP) | Ptafr-Rn02132919_s1 |
|  | Ptgdr | Prostaglandin D2 receptor (DP) | Ptgdr-Rn00824628_m1 |
|  | Ptger2 | Prostaglandin E receptor 2 (subtype EP2), 53kDa | Ptger2-Rn00579419_m1 |
|  | Ptger3 | Prostaglandin E receptor 3 (subtype EP3) | Ptger3-Rn00562282_m1 |
|  | Ptgfr | Prostaglandin F receptor (FP) | Ptgfr-Rn00565423_m1 |
|  | Ptgir | Prostaglandin I2 (prostacyclin) receptor (IP) | Ptgir-Rn01764022_m1 |
|  | Ptgis | Prostaglandin I2 (prostacyclin) synthase | Ptgis-Rn00694611_m1 |
|  | Ptgs1 | Prostaglandin-endoperoxide synthase 1 | Ptgs1-Rn00566881_m1 |
|  | Ptgs2 | Prostaglandin-endoperoxide synthase 2 | Ptgs2-Rn01483828_m1 |
|  | Scye1 | Small inducible cytokine subfamily E, member 1 | Scye1-Rn01518601_m1 |
|  | Tbxa2r | Thromboxane A2 receptor | Tbxa2r-Rn00690601_m1 |
|  | Tbxas1 | Thromboxane A synthase 1 (platelet) | Tbxas1-Rn00562160_m1 |
|  | Tlr2 | Toll-like receptor 2 | Tlr2-Rn02133647_s1 |
|  | Tlr4 | Toll-like receptor 4 | Tlr4-Rn00569848_m1 |
|  | Tnf | Tumor necrosis factor (TNF superfamily, member 2) | Tnf-Rn99999017_m1 |
|  | Tnfrsf1a | Tumor necrosis factor receptor superfamily, member 1A | Tnfrsf1a-Rn01492348_m1 |
|  | Tnfrsf1b | Tumor necrosis factor receptor superfamily, member 1A | Tnfrsf1b-Rn00709830_m1 |
|  | Vcam1 | Vascular cell adhesion molecule 1 | Vcam1-Rn00563627_m1 |
| Estrogen & Progesterone Receptors | Esr1 | Estrogen receptor 1 (ER alpha) | Esr1-Rn01640372_m1 |
|  | Esr2 | Estrogen receptor 2 (ER beta) | Esr2-Rn00562610_m1 |
|  | Esrra | Estrogen-related receptor alpha | Esrra-Rn00433142_m1 |
|  | Esrrb | Estrogen-related receptor beta | Esrrb-Rn02606541_m1 |
|  | Esrrg | Estrogen-related receptor gamma | Esrrg-Rn01415309_m1 |
|  | Gper | G protein-coupled estrogen receptor 1 | Gper-Rn01643280_s1 |
|  | Pgr | Progesterone receptor | Pgr-Rn01448227_m1 |
|  | Pgrmc1 | Progesterone receptor membrane component 1 | Pgrmc1-Rn01774803_m1 |
|  | Pgrmc2 | Progesterone receptor membrane component 2 | Pgrmc2-Rn01755753_m1 |
| Control genes | 18S | 18S ribosomal RNA | 18S-Hs99999901_s1 |
|  | Actb | Actin, beta | Actb-Rn00667869_m1 |
|  | Gapdh | Glyceraldehyde-3-phosphate dehydrogenase | Gapdh-Rn99999916_s1 |
|  | Hprt1 | Hypoxanthine phosphoribosyltransferase 1 | Hprt1-Rn01527840_m1 |

Genes colored in grey were undetermined for at least one of two reasons: 1) no amplification possibly due to low expression or failed assays; 2) two or fewer samples with undetectable Ct values within any of the seven groups.
