# Supplementary material for: Continuous versus Cyclic Progesterone Exposure Differentially Regulates Hippocampal Gene Expression and Functional Profiles
Source: PLoS One. 2012 Feb 29;7(2):e31267. doi: 10.1371/journal.pone.0031267 (PMC3290616; doi:10.1371/journal.pone.0031267)
Supplement: Table S3 — Gene expression changes in response to different hormone interventions and treatment paradigms. (DOCX) [file pone.0031267.s003.docx]

**Table S3:** Gene expression changes in response to different hormone interventions and treatment paradigms.

| Functional Group | Gene Symbol | Gene Expression (Compared to OVX) | | | | | | | | | | | |
| --- | --- | --- | --- | --- | --- | --- | --- | --- | --- | --- | --- | --- | --- |
|  |  | Sham-OVX | | OVX+E2 | | OVX+CoP4 | | OVX+CyP4 | | OVX+E2+CoP4 | | OVX+E2+CyP4 | |
|  |  | Fold Change | P-Value | Fold Change | P-Value | Fold Change | P-Value | Fold Change | P-Value | Fold Change | P-Value | Fold Change | P-Value |
| Mitochondrial Energy & Redox Metabolism | **Atp5a1** | **1.46** | **0.044*** | 1.33 | 0.598 | 1.10 | 0.668 | **1.58** | **0.020*** | 0.78 | 0.300 | **1.91** | **0.046*** |
|  | Cox4i1 | 1.45 | 0.127 | 1.26 | 0.646 | 1.09 | 0.761 | 1.48 | 0.107 | 0.81 | 0.464 | 1.80 | 0.064 |
|  | **Dnm1l** | 1.00 | 0.979 | 0.93 | 0.484 | 0.93 | 0.390 | 1.07 | 0.492 | **0.78** | **0.004**** | 1.08 | 0.252 |
|  | **Hadh** | 1.04 | 0.740 | 0.86 | 0.284 | 0.91 | 0.403 | 1.20 | 0.256 | **0.69** | **0.021*** | 1.18 | 0.177 |
|  | **Mfn1** | 0.77 | 0.099 | 0.75 | 0.084 | **0.66** | **0.037*** | 0.78 | 0.146 | **0.68** | **0.038*** | 0.80 | 0.143 |
|  | **Mfn2** | 0.92 | 0.337 | 0.92 | 0.479 | 0.79 | 0.084 | 0.85 | 0.241 | 0.88 | 0.134 | **0.81** | **0.022*** |
|  | Nfe2l2 | 0.96 | 0.735 | 0.81 | 0.060 | 0.93 | 0.532 | 0.87 | 0.344 | 0.76 | 0.131 | 1.01 | 0.894 |
|  | Nrf1 | 0.93 | 0.589 | 0.92 | 0.539 | 0.71 | 0.069 | 0.93 | 0.604 | 0.77 | 0.132 | 0.90 | 0.513 |
|  | **Pdha1** | 0.85 | 0.095 | 0.86 | 0.104 | 0.87 | 0.163 | 0.92 | 0.551 | **0.81** | **0.042*** | 0.93 | 0.406 |
|  | Pdhb | 1.20 | 0.156 | 1.11 | 0.617 | 1.04 | 0.783 | 1.38 | 0.150 | 0.96 | 0.702 | 1.37 | 0.090 |
|  | **Polg** | **0.63** | **0.012*** | 0.85 | 0.284 | **0.67** | **0.027*** | **0.61** | **0.014*** | 0.82 | 0.270 | **0.64** | **0.007**** |
|  | **Ppargc1b** | 0.70 | 0.059 | 0.90 | 0.687 | **0.68** | **0.048*** | 0.65 | 0.053 | 0.72 | 0.059 | **0.63** | **0.025*** |
|  | **Prdx5** | **1.28** | **0.047*** | 1.09 | 0.652 | 1.12 | 0.458 | 1.19 | 0.185 | **0.76** | **0.043*** | **1.34** | **0.009**** |
|  | **Sirt1** | 1.30 | 0.083 | 1.20 | 0.087 | 0.96 | 0.696 | 1.32 | 0.078 | 0.96 | 0.733 | **1.32** | **0.021*** |
|  | **Slc25a4** | 1.34 | 0.122 | 1.23 | 0.690 | 1.12 | 0.621 | 1.65 | 0.056 | 0.82 | 0.397 | **1.77** | **0.037*** |
|  | **Slc2a3** | 0.85 | 0.256 | 0.83 | 0.152 | 0.81 | 0.104 | 0.75 | 0.066 | 0.78 | 0.071 | **0.69** | **0.029*** |
|  | **Sod2** | **0.82** | **0.028*** | 0.94 | 0.568 | 0.91 | 0.189 | **0.85** | **0.023*** | 0.90 | 0.073 | 0.91 | 0.166 |
|  | Tfam | 1.14 | 0.080 | 1.05 | 0.825 | 0.93 | 0.538 | 1.29 | 0.142 | 0.81 | 0.076 | 1.30 | 0.065 |
| Cholesterol Homeostasis & Myelin Metabolism | **Apoe** | 0.94 | 0.463 | **0.77** | **0.015*** | 1.06 | 0.598 | 1.04 | 0.668 | **0.89** | **0.033*** | 1.07 | 0.233 |
|  | Capn1 | 1.22 | 0.237 | 0.89 | 0.528 | 0.76 | 0.245 | 1.02 | 0.935 | 0.64 | 0.060 | 0.96 | 0.771 |
|  | **Cd81** | 0.95 | 0.511 | **0.76** | **0.046*** | 1.01 | 0.931 | 1.02 | 0.879 | 0.81 | 0.064 | 1.08 | 0.466 |
|  | **Ctsb** | 1.05 | 0.397 | 0.96 | 0.663 | 1.03 | 0774 | 1.01 | 0.960 | **0.83** | **0.031*** | 1.11 | 0.276 |
|  | **Cyp27a1** | 0.85 | 0.644 | 0.93 | 0.776 | **0.53** | **0.046*** | 0.76 | 0.494 | 0.63 | 0.105 | 1.20 | 0.540 |
|  | **Galc** | 1.08 | 0.420 | **0.70** | **4.00E-4***** | **0.78** | **0.049*** | 1.09 | 0.599 | **0.68** | **5.00E-4***** | 1.13 | 0.212 |
|  | **Nr1h3** | **1.98** | **0.031*** | 1.35 | 0.361 | 1.54 | 0.087 | 1.47 | 0.307 | 0.85 | 0.561 | **2.07** | **0.035*** |
|  | Smpd1 | 1.22 | 0.059 | 0.99 | 0.936 | 1.16 | 0.405 | 1.20 | 0.378 | 0.85 | 0.159 | 1.17 | 0.106 |
|  | Tspo | 1.37 | 0.115 | 0.86 | 0.353 | 0.88 | 0.343 | 1.23 | 0.270 | 0.70 | 0.060 | 1.75 | 0.273 |
| Insulin Signaling & Amyloid Metabolism | Adam17 | 0.84 | 0.284 | 0.67 | 0.072 | 0.75 | 0.137 | 0.71 | 0.081 | 0.72 | 0.089 | 0.83 | 0.327 |
|  | **Apba1** | 0.83 | 0.144 | 0.84 | 0.276 | 0.80 | 0.153 | 0.79 | 0.106 | 0.85 | 0.183 | **0.65** | **0.013*** |
|  | **Apba2** | 0.91 | 0.263 | 0.85 | 0.094 | **0.79** | **0.011*** | 0.82 | 0.052 | 0.90 | 0.074 | 0.79 | 0.065 |
|  | **Apbb1** | 0.93 | 0.551 | 0.92 | 0.618 | 0.92 | 0.462 | 0.92 | 0.607 | 0.94 | 0.581 | **0.72** | **0.020*** |
|  | Apbb2 | 0.96 | 0.764 | 0.88 | 0.355 | 0.84 | 0.280 | 0.85 | 0.315 | 0.72 | 0.081 | 0.83 | 0.234 |
|  | Apbb3 | 1.23 | 0.077 | 1.16 | 0.283 | 1.05 | 0.707 | 1.21 | 0.193 | 1.08 | 0.602 | 1.02 | 0.856 |
|  | Apeh | 0.83 | 0.067 | 0.93 | 0.709 | 0.84 | 0.056 | 0.95 | 0.780 | 1.02 | 0.893 | 0.83 | 0.162 |
|  | **Bace1** | **0.77** | **0.033*** | 0.90 | 0.664 | 0.79 | 0.088 | **0.77** | **0.031*** | 0.90 | 0.462 | **0.57** | **9.00E-04***** |
|  | **Igf1** | 1.76 | 0.053 | 1.04 | 0.903 | 1.34 | 0.209 | **1.83** | **0.019*** | 0.89 | 0.677 | **1.95** | **0.008**** |
|  | Nae1 | 1.17 | 0.065 | 1.26 | 0.401 | 0.97 | 0.808 | 1.28 | 0.078 | 0.90 | 0.403 | 1.42 | 0.099 |
|  | **Ncstn** | 1.04 | 0.763 | 0.95 | 0.606 | **0.83** | **0.023*** | 0.96 | 0.685 | 0.87 | 0.184 | 0.94 | 0.569 |
|  | Prep | 1.05 | 0.618 | 0.94 | 0.526 | 0.94 | 0.559 | 1.02 | 0.883 | 1.04 | 0.716 | 0.83 | 0.092 |
|  | Psen1 | 0.75 | 0.091 | 0.82 | 0.181 | 0.75 | 0.088 | 0.85 | 0.304 | 0.86 | 0.373 | 0.79 | 0.127 |
|  | **Psen2** | 0.87 | 0.229 | 0.95 | 0.774 | 0.81 | 0.182 | 0.76 | 0.179 | 0.85 | 0.157 | **0.76** | **0.049*** |
|  | Psenen | 1.64 | 0.057 | 1.43 | 0.533 | 1.09 | 0.775 | 1.64 | 0.060 | 0.81 | 0.492 | 1.93 | 0.068 |
| Inflammation | **Adrb1** | **0.71** | **0.044*** | 0.45 | 0.206 | **0.58** | **0.026*** | 0.76 | 0.278 | 0.97 | 0.889 | 0.71 | 0.131 |
|  | Adrb2 | 0.60 | 0.202 | 1.23 | 0.517 | 0.70 | 0.172 | 0.55 | 0.092 | 0.71 | 0.249 | 0.77 | 0.280 |
|  | Alox12 | 0.33 | 0.054 | 0.67 | 0.199 | 0.56 | 0.167 | 0.49 | 0.127 | 0.40 | 0.057 | 0.75 | 0.558 |
|  | Alox5 | 1.41 | 0.304 | 1.49 | 0.281 | 1.26 | 0.506 | 1.37 | 0.370 | 1.79 | 0.070 | 1.04 | 0.809 |
|  | Anxa3 | 0.86 | 0.165 | 0.79 | 0.525 | 0.84 | 0.324 | 0.92 | 0.778 | 0.78 | 0.060 | 1.11 | 0.531 |
|  | **C1qb** | **0.71** | **0.027*** | 0.74 | 0.133 | 0.88 | 0.409 | **0.70** | **0.038*** | 0.91 | 0.397 | 0.74 | 0.055 |
|  | **Casp1** | 1.05 | 0.963 | **0.31** | **0.034*** | 0.76 | 0.406 | 1.04 | 0.907 | 0.75 | 0.338 | 1.38 | 0.436 |
|  | Cysltr1 | 0.72 | 0.143 | 0.97 | 0.723 | 0.96 | 0.832 | 0.64 | 0.233 | 0.69 | 0.079 | 0.84 | 0.374 |
|  | **Hrh2** | 0.83 | 0.061 | 1.29 | 0.239 | **0.71** | **0.037*** | 0.83 | 0.487 | 0.89 | 0.264 | 0.84 | 0.575 |
|  | Hrh3 | 0.70 | 0.208 | 0.68 | 0.246 | 0.67 | 0.206 | 0.55 | 0.089 | 0.96 | 0.751 | 0.63 | 0.134 |
|  | **Icam1** | 0.84 | 0.432 | 0.81 | 0.609 | 0.61 | 0.212 | 0.80 | 0.328 | 0.79 | 0.994 | **0.35** | **0.040*** |
|  | **Il1rapl2** | **0.18** | **0.040*** | 0.57 | 0.138 | 0.40 | 0.079 | 0.62 | 0.193 | 0.50 | 0.134 | 0.31 | 0.088 |
|  | **Itgal** | **0.47** | **0.007**** | 0.68 | 0.095 | **0.67** | **0.047*** | 0.80 | 0.166 | 0.81 | 0.478 | 0.75 | 0.395 |
|  | **Itgam** | 0.81 | 0.157 | **0.60** | **0.004**** | 0.85 | 0.169 | **0.70** | **0.009**** | **0.76** | **0.049*** | 0.78 | 0.286 |
|  | Itgb1 | 0.80 | 0.242 | 0.78 | 0.345 | 0.70 | 0.066 | 0.73 | 0.128 | 0.77 | 0.143 | 0.78 | 0.101 |
|  | Lta4h | 1.21 | 0.315 | 0.25 | 0.073 | 0.87 | 0.668 | 1.25 | 0.259 | 0.83 | 0.372 | 1.53 | 0.189 |
|  | **Mapk1** | 0.99 | 0.917 | 0.96 | 0.865 | **0.81** | **0.046*** | 1.00 | 0.872 | 0.98 | 0.834 | 0.95 | 0.822 |
|  | **Mapk14** | 0.94 | 0.553 | 1.04 | 0.623 | **0.77** | **0.005**** | 0.89 | 0.717 | 0.97 | 0.944 | 0.83 | 0.401 |
|  | **Mapk8** | 0.66 | 0.069 | 0.69 | 0.079 | **0.56** | **0.035*** | 0.69 | 0.099 | 0.80 | 0.203 | 0.80 | 0.274 |
|  | **Nfkb1** | 0.95 | 0.618 | 0.85 | 0.363 | **0.70** | **0.033*** | 0.75 | 0.283 | **0.71** | **0.016*** | 1.07 | 0.632 |
|  | **Nr3c1** | 0.88 | 0.395 | **0.81** | **0.033*** | 0.84 | 0.349 | 1.02 | 0.793 | 0.79 | 0.078 | 0.94 | 0.751 |
|  | Pde4b | 1.01 | 0.869 | 1.14 | 0.396 | 0.88 | 0.091 | 0.85 | 0.381 | 0.97 | 0.778 | 1.08 | 0.525 |
|  | Pde4d | 0.87 | 0.344 | 0.79 | 0.108 | 0.78 | 0.138 | 0.91 | 0.734 | 0.80 | 0.070 | 0.86 | 0.304 |
|  | **Pla2g1b** | 1.34 | 0.377 | 0.87 | 0.845 | **0.47** | **0.020*** | 0.79 | 0.978 | 0.72 | 0.355 | 1.40 | 0.340 |
|  | **Pla2g2a** | **0.28** | **0.044*** | 0.60 | 0.184 | 0.51 | 0.157 | 0.80 | 0.520 | 0.70 | 0.3388 | 1.22 | 0.698 |
|  | **Plcb4** | 0.91 | 0.561 | 1.15 | 0.507 | 0.84 | 0.594 | **0.72** | **0.013*** | **0.64** | **0.006**** | 1.08 | 0.542 |
|  | Plcd1 | 0.74 | 0.147 | 0.67 | 0.055 | 0.79 | 0.122 | 0.82 | 0.234 | 0.75 | 0.059 | 0.81 | 0.280 |
|  | **Plcg1** | 0.74 | 0.162 | 0.72 | 0.266 | **0.50** | **0.021*** | 0.67 | 0.152 | 0.82 | 0.264 | 0.60 | 0.056 |
|  | **Plcg2** | 0.70 | 0.058 | **0.45** | **0.019*** | **0.46** | **0.017*** | 0.71 | 0.099 | 0.87 | 0.601 | **0.57** | **0.042*** |
|  | **Ptafr** | 0.97 | 0.947 | **0.26** | **0.009**** | 0.71 | 0.603 | 1.14 | 0.508 | 0.78 | 0.152 | 1.33 | 0.305 |
|  | Ptger3 | 1.52 | 0.148 | 0.97 | 0.748 | 1.28 | 0.128 | 1.79 | 0.177 | 1.41 | 0.215 | 1.82 | 0.098 |
|  | Ptgis | 0.66 | 0.559 | 1.35 | 0.513 | 0.30 | 0.073 | 1.35 | 0.847 | 0.93 | 0.897 | 1.01 | 0.926 |
|  | Scye1 | 1.17 | 0.301 | 0.98 | 0.849 | 0.72 | 0.114 | 1.13 | 0.442 | 0.86 | 0.301 | 1.51 | 0.066 |
|  | Tlr2 | 1.00 | 0.807 | 0.37 | 0.073 | 0.96 | 0.927 | 1.29 | 0.388 | 0.74 | 0.320 | 1.39 | 0.306 |
|  | **Tnfrsf1b** | 0.90 | 0.834 | 0.65 | 0.156 | 0.77 | 0.105 | **0.64** | **0.049*** | 0.97 | 0.853 | 0.68 | 0.063 |
|  | **Vcam1** | 0.92 | 0.626 | 0.84 | 0.292 | 0.84 | 0.163 | 0.96 | 0.984 | **0.77** | **0.005**** | 1.01 | 0.820 |
| Estrogen & Progesterone Receptors | **Pgr** | 0.80 | 0.067 | **0.75** | **0.009**** | **0.76** | **0.011*** | 0.79 | 0.066 | **0.73** | **0.005**** | 0.86 | 0.216 |
|  | **Pgrmc1** | 1.24 | 0.174 | 1.11 | 0.682 | 1.11 | 0.215 | **1.26** | **0.028*** | 0.94 | 0.486 | **1.49** | **0.037*** |

This table lists genes that exhibited expression changes with P < 0.1 (compared to the OVX control group); genes with P < 0.05 are highlighted in **bold**. **Red**: up-expression (* P < 0.05; ** P < 0.01; *** P < 0.001); **Green**: down-expression (* P < 0.05; ** P < 0.01; *** P < 0.001).
